# Design of Rural Human Resource Management Platform Integrating IoT and Cloud Computing

**DOI:** 10.1155/2022/4133048

**Published:** 2022-04-30

**Authors:** Meng Chai

**Affiliations:** Political Science and Public Administration, Guangxi University for Nationalities, Nanning, Guangxi 53000, China

## Abstract

With the advent of the Internet of Things era, these hot technologies such as distributed, parallel computing, network storage, and load balancing can provide a good application foundation for the Internet of Things, enabling real-time dynamic management and intelligent analysis of hundreds of millions of items in the Internet of Things to be possible. The Internet of Things has changed from a concept to a reality, quickly reaching every corner of society. On the other hand, with the enhancement of the mobility of social talents, the file management of the talent service center is becoming more and more difficult. However, the traditional management methods of human resources files have problems such as poor resource sharing, asymmetric resources, and heterogeneous information sharing, which can no longer meet the needs of both the supply and demand sides of human resources with diversified and multiple organizational structures. Cloud computing technology has powerful data collection functions, self-service functions, and unified resource scheduling functions. Introducing it into the human resources file management system can greatly improve management efficiency. In order to carry out information management of rural human resources, this paper develops a rural human resources management system based on the Internet of Things. This paper introduces the design scheme of rural human resource management platform based on Internet of Things technology and cloud computing technology. The design of this system mainly includes organization setting, post planning, personnel management, salary management, insurance benefits, recruitment and selection, training management, performance appraisal management, labor contract management, comprehensive inquiry, rules and regulations, employee self-help, system setting, and system management function modules. The research results show that the rural human resource management system based on cloud computing can provide a complete human resource management solution for the vast rural areas. It can only purchase services, save a lot of development and maintenance costs, and also customize functions, so as to better meet the needs of use.

## 1. Introduction

The management of human resources refers to the use of modern science and technology and human resource management theories, in order to achieve the strategic goals of the organization, by continuously acquiring human resources from the society and adjusting, integrating, and developing the acquired human resources [1–5].

Along with the global economic integration and the formation of the “global village”, in the 21st century knowledge economy era with knowledge as the theme, rural areas are facing increasing competition, and the main job of the competition lies in the competition of talents, but also in the digging of the maximum potential of one's own talents. How to optimize the combination of talents and give full play to the advantages of human resources has become the biggest challenge faced by rural areas and the main factor for the existence and development of rural areas. The whole system of this research project is divided into three-tier structure. The three-tier architecture divides the whole system into three layers, including presentation layer, business logic layer, and data persistence layer.

The presentation layer involves interacting with the user, presentation, and functionality designed to validate input data. The presentation layer is the main way for the system to provide GUI interaction to the user: the user can input data or edit the existing data: verify the data entered or edited by the user by validating the data [6–10].

The business logic layer has a certain role to undertake, which is located between the data presentation layer and the access layer. The layer belongs to the category of weakly coupled structure, there is a certain dependency between the layers, and for the upper layer, the lower layer is unknown. Therefore, certain adjustments to the upper-layer design will not be called for the bottom layer causing an impact. If the layered design is based on the interface-oriented design idea, the corresponding downward dependency also belongs to the category of weak dependencies, which means that the business logic layer integrates the dependencies. For personnel, they should not only pay attention to the realization of business logic, but also pay attention to the realization of decoupling of dependencies [11–14].

The above three-layer software design scheme for rural application system will greatly improve the reusability and expandability of the system. On the one hand, this system can realize a more rational and effective application of rural resource allocation strategies; on the other hand, it can also help improve software performance indicators, ensure system security, and provide convenience for management. From the point of view of software design and development, design pattern is a revolutionary achievement of great significance in software development. It is the effective crystallization of software design in the specific practice process and makes many seemingly tedious problems simple and clear. The design pattern has the following five functions in the process of this system development. (1) Code reuse design: Code reuse design is more practical than the original reuse code, it can make code reuse, and its utility is stronger. (2) Providing common vocabulary for design: Each pattern name corresponds to a corresponding design vocabulary, and the simplification of pattern concepts can provide a more relaxed environment for programmers to communicate. (3) Frequent communication between designers: The use of certain pattern vocabulary in the specific development of relevant documents can improve the understanding of relevant personnel; it is also easier to write development documents. (4) It is easier to refactor the system, the correctness of its code development is guaranteed, and the degree of errors in specific design or implementation can be reduced. (5) Efficient and rational system architecture: The rational use of design patterns can greatly save time and improve efficiency [15–21].

The human resource management system includes a wide range of content, involving the management of rural personnel information, employee recruitment, performance appraisal, attendance management, salary management, etc. Before the popularization of information technology, human resource management mainly relied on the form of paper files, low efficiency. With the popularization and application of computer technology, almost all rural areas use computer systems to manage human resource management business. Especially after the emergence of the Internet, human resource management has entered the stage of resource sharing and remote data transmission, increasing day by day [21].

In order to better meet the application needs of the rural areas, a knowledge-based human resource management system has emerged, which is characterized by the analysis of a large amount of human resource management information to obtain the auxiliary decision-making information required by the rural management. There are two main forms of design and development of rural human resource management system. One is to purchase commercial software. The advantage is that the development cycle is short, and it can be used immediately. Entrusting software companies to develop, the advantage is that it can be modified and customized, but the disadvantage is that the development cycle is long, the investment is large, and the later maintenance is inconvenient.

Since 1950, countries around the world have been scrambling to develop artificial intelligence machines. When the program has certain judgment standards and systems, it can be used to replace human resources in certain situations, which is what we call AI today (artificial intelligence) and cognitive computing. In 1980, after everyone had a general understanding of the definition of artificial intelligence, this milestone attempt was suspended due to the limitations of technological development. Today, with the rapid development of high-tech, the continuous innovation of mechanical components, and the cutting-edge research of computer science, many emerging technologies can be integrated into a single mechanical entity to realize the replacement of hardware devices. Due to the continuous innovation of computer technology and the integration of multidisciplinary and multifield technologies, we can see the application of deep learning everywhere in our daily life, such as face recognition, recommendation systems, and so on.

## 2. IoT and Cloud Computing

**Figure 1:**
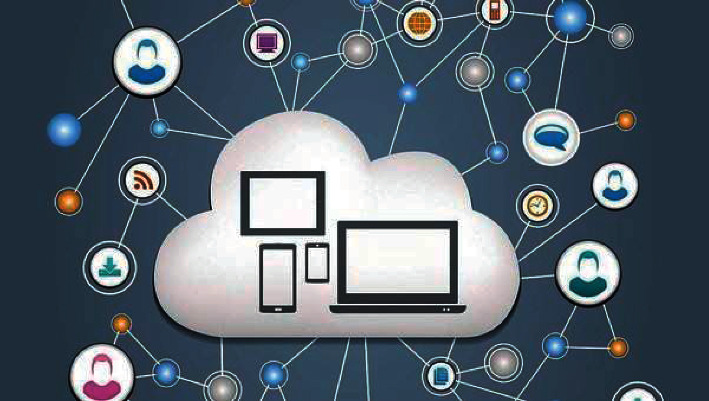
Cloud computing.

**Figure 2:**
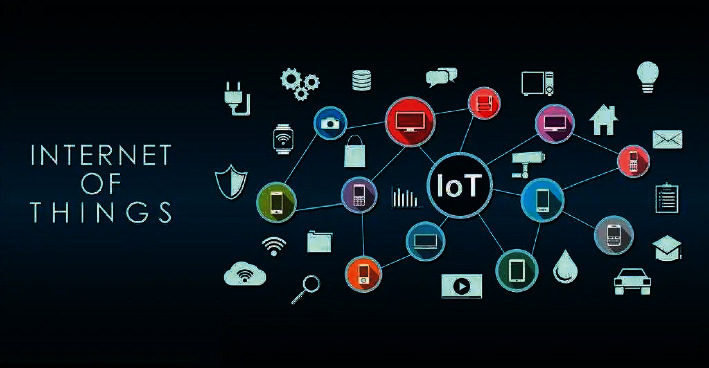
Internet of Things.

Cloud computing (see Figure 1) and the Internet of Things (see Figure 2) are two rapidly developing new technologies. The ultra-large scale, virtualization, high reliability, and good scalability of cloud computing are exactly the technologies required for the large-scale and intelligent development of the Internet of Things. Software cloudification is an application model that provides software services based on cloud computing networks. It has good flexibility, adaptability, and scalability. These characteristics are very suitable for the Internet of Things. The efficient computing mode of cloud computing can provide a good application foundation for the Internet of Things, making real-time dynamic management and intelligent analysis of hundreds of millions of items possible. The innovative service delivery model of cloud computing strengthens the interconnection between the Internet of Things and the Internet and also accelerates the intelligent integration of the Internet of Things and cloud computing.

At present, with the development of cloud computing technology, the Internet of Things is involved in our lives in many fields such as smart home, smart logistics, smart grid, smart medical care, and smart transportation, as well as smart city and ecological monitoring. The Internet of Things will play an important role in transforming traditional industries, leading emerging industries, improving people's lives, and providing national security. Forrester, an authoritative market research organization in the United States, once predicted that, by 2020, the total output value of the Internet of Things industry will be 30 times that of mobile terminals and the Internet combined, and the broad development prospects of the Internet of Things will have a huge impact on the economy and society. The annual blue book of China's Internet of Things industry development also predicts that, in 2015, the global Internet of Things industry will reach a market size of 350 billion US dollars. From this, it is not difficult to speculate that, in the next 10 years, the Internet of Things industry will achieve large-scale popularity in the world.

At present, human resource management can use new technologies to gradually upgrade on the basis of the development of the original traditional human resources and digital human resources and integrate the Internet of Things technology and cloud computing technology into the human resources management platform to realize a smart human resources museum. Smart human resource is an intelligent management of diversified human resources based on new technologies such as the Internet of Things and cloud computing. The management object of smart human resources is diversified human resources, including the content information and carrier information of traditional human resources and new human resources. The intelligent human resource management platform has the advantages of being fast, efficient, accurate, and safe in the analysis of human resource information resources and management.

The Internet of Things is to interconnect all items with the Internet through radio frequency identification, infrared sensors.

The Internet of Things includes a perception layer, a network layer, and an application layer.

1. Perception layer is located at the bottom of the three-layer structure of the Internet of Things. The perception layer is necessary to realize the information collection, capture, and identification of objects. Information collection mainly adopts radio frequency identification technology and sensor technology. Radio frequency identification technology first places a tag on the object and reads the information in the tag through the proximity activity between the identification device and the tag, so as to automatically obtain the relevant information of the identified object; the sensor is a kind of physical quantity or chemical quantity that can be converted into electricity. The signal device can sense the surrounding temperature, speed, electromagnetic radiation or gas composition, etc. and is mainly used to collect various information around the sensor.
2. Network layer: the network layer belongs to the middle layer in the three-layer structure of the Internet of Things, and its main task is to realize the transmission of information. The network layer is responsible for accurately conveying various sensing data accessed by the sensing layer to the application layer. The transmission of information constitutes the basis for the interconnection of things and people. Due to its complexity, the communication of the Internet of Things includes almost all existing communication technologies. The network layer includes two types of access network and core network. The access network mainly provides network access and mobility management functions for terminal equipment. The core network is a high-performance and scalable network based on the telecommunication network and the Internet and supports various heterogeneous access methods.
3. Application layer: the application layer is located at the top of the three-layer structure of the Internet of Things, which realizes the rapid storage of information, intelligent analysis of data, and decision support for applications and realizes various intelligent applications of the Internet of Things. In the process of intelligent analysis of applications, cloud computing is the core technology. The high-efficiency computing mode of cloud computing, such as distributed, parallel computing, network storage, load balancing, middleware, and other technologies, can provide a good application foundation for the application layer system. With the rapid development of IoT applications and a large increase in the number of terminals, cloud computing is required to process big data information and provide users with final decision support through intelligent analysis.

With the rapid development of Internet technology, the amount of information generated in people's production and life shows an explosive growth trend. In recent years, with the explosive development of social networks and video networks, the type of network data has gradually shifted from traditional text data to images, audio, and video. The high-precision processing of this part of the network data has become the focus and difficulty in the current big data research. How to effectively search and cluster analysis on big data has always been an important research point. The core content of the huge value behind the data to be mined is to choose the appropriate processing method. In the face of big data and network equipment, choosing new technologies is the main way to solve the current difficulties in data analysis.

## 3. Design of Parallel Search Clustering Algorithm for Big Data Based on Cloud Computing

In this study, the word segmentation dictionary is applied to the data search process. Set the current state of the data as *y* and the next data of the current data as *j*, the data state is transferred from *y* to the state yg, if the next data of the current data is *s*, then transfer to the data state ys, and then this part of the operation should satisfy the following requirements:

(1) $$\begin{array}{c} \text{base}\left[y\right]+j=\text{base}\left[\text{yg}\right],\\ \text{base}\left[y\right]+s=\text{base}\left[\text{ys}\right],\\ \text{check}\left[\text{ys}\right]=\text{check}\left[\text{yg}\right]=\text{base}\left[y\right]. \end{array}$$

In order to ensure the use effect, the above calculation process is controlled by the average degree of data length. The specific formula is as follows:

(2) $$\begin{array}{c} \text{LenAgg}=\prod_{i=1}^{n}\text{Len}\left(a_{i}\right),\\ \text{LenWeight}=\prod_{i=1}^{n}i*\text{Len}\left(a_{i}\right). \end{array}$$

The above formula is used to control the data search process to ensure that the data search and retrieval can be carried out simultaneously with the big data clustering process. Similarity is comparing the similarity of two things. Generally, by calculating the distance between the features of things, if the distance is small, the similarity is large; if the distance is large, the similarity is small. The smaller the similarity, the stronger the clustering ability. In this study, the clustering ability of the current algorithm is optimized. Clustering is performed according to the nearest principle, and Euclidean distance is used to calculate the distance between network data. The specific formula is as follows:

(3) $$\begin{array}{c} d\left(a,b\right)=\sqrt{{\left(c_{a1}-c_{b1}\right)}^{2}+{\left(c_{a2}-c_{b2}\right)}^{2}+\cdots +{\left(c_{\text{an}}-c_{\text{bn}}\right)}^{2}}, \end{array}$$

where *c*_*a*_ and *c*_*b*_ represent two n-dimensional network data objects. If the weight of each dimension is different in the data processing process, the attributes of each dimension need to be weighted to obtain the weighted data distance. The specific calculation formula is as follows:

(4) $$\begin{array}{c} d\left(a,b\right)=\sqrt{k_{1}{\left(c_{a1}-c_{b1}\right)}^{2}+k_{2}{\left(c_{a2}-c_{b2}\right)}^{2}+\cdots +k_{3}{\left(c_{\text{an}}-c_{\text{bn}}\right)}^{2}}. \end{array}$$

The above formula is used to calculate the similarity and cluster analysis of the network data, and the absolute error criterion is used to verify the calculation results of this formula. According to the requirements of network data usage, the absolute error criterion is defined as follows:

(5) $$\begin{array}{c} H=\sum_{i=1}^{k}{\sum_{c\in F}\left\|c_{a}-p_{i}\right\|}^{2}, \end{array}$$

where H represents the absolute squared error sum of all data in the network database, p1, p2,…, pi,…, pn represent, from the nth data cluster F1, F2,…, the representative reference point selected by Fn. According to the above formula, the network data cluster analysis result can be obtained, and the data cluster analysis process is completed.

In the above, the search process and clustering analysis of big data have been designed accordingly. In this section, cloud computing technology is used to set up the parallel processing part of big data, so as to complete the design of the clustering algorithm for parallel search of big data process. The design and use effect of the parallel process in the current algorithm conforms to the current data requirements, but it is less restrictive. To this end, the constraints in the parallel processing process are set in this study. The specific constraints are as follows:

Calculating time:

(6) $$\begin{array}{c} T=\frac{E*Q}{U}, \end{array}$$

where *T* represents the computing time, *E* represents the amount of information in the data, *Q* represents the length of the data query sequence, and *U* represents the data cluster analysis capability.

Data read speed:

(7) $$\begin{array}{c} f\left(s\right)=\frac{E}{T}, \end{array}$$

where *f* (*s*) represents the database reading speed. Substituting formula (7) into formula (6), the deformation formula of database reading speed can be obtained, as follows:

(8) $$\begin{array}{c} f\left(s\right)=\frac{U}{T}. \end{array}$$

Use this formula to scale and control data read speed.

**Figure 3:**
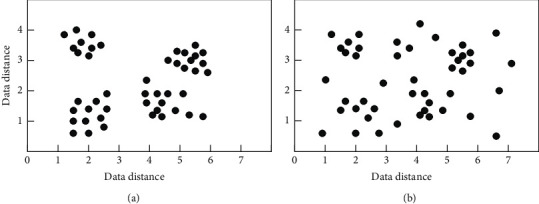
Comparison. (a) Proposed method. (b) Conventional method.

Database classification:

(9) $$\begin{array}{c} K_{i}=\frac{L_{i}}{\sum L_{i}}*O, \end{array}$$

where *i* represents the data node, *L*_*i*_ represents the running performance of the database, and O represents the size of the database. Use the above formula to control the parallel processing of big data and realize the parallel search and cluster analysis process of data. So far, the design of the big data parallel search clustering algorithm based on cloud computing is completed. The data is compared in Figure 3.

## 4. System Design

**Figure 4:**
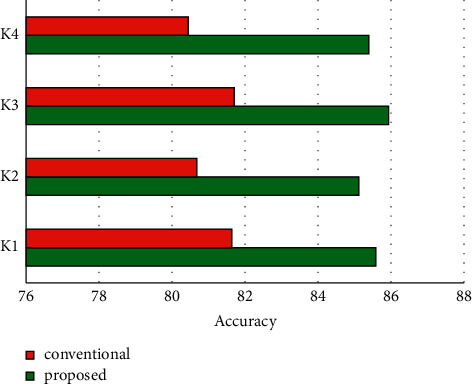
Accuracy of data item.

The human resource information management system based on “cloud computing” collects data through keyboards, two-dimensional codes, bar codes, etc. and then standardizes various types of data in it, so as to realize the concentration of data and the sharing of resources. “Cloud Computing” public service center: The system architecture includes 5 layers including basic data collection layer, network service support layer, cloud computing support layer, data standardization conversion layer, and system application layer. The basic data collection layer enters various types of data into the system according to the specified format; the network service support layer realizes the construction of an information collection network based on hybrid convergence points; the cloud computing support layer transfers the data transmitted from the network service support layer to the “cloud” in order to realize the sharing of resources and the cloud processing of data; the data standardization conversion layer standardizes the data transmitted from the “cloud,” establishes a unified description of the multidimensional human resource file data view model, and supports unified access of various components of system. A dedicated middleware for integrating data: The system application layer provides users with services such as human resource file maintenance, human resource file management authorization maintenance index, and information resource integration in B/S mode. The accuracy of data item is compared in Figure 4.

**Figure 5:**
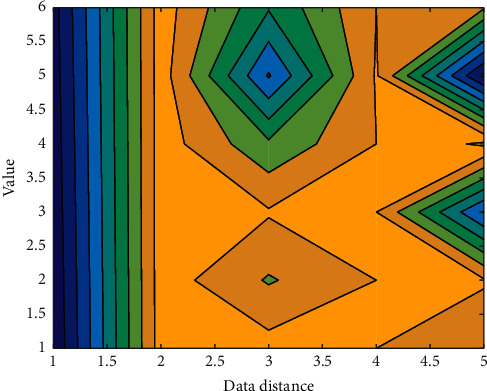
Value.

The overall design goal of the rural human resource management system based on cloud computing is to build a set of cloud computing platform-based human resources for the current large group-type rural areas that lack the ability to handle massive human resources and the inconvenience of maintaining the human resource management system in use. The resource management system makes full use of the characteristics of cloud computing software-as-a-service, provides the required services for rural areas by renting, and uses the parallel computing model to solve the problem of sharing massive human resource data. The specific design goals of the system include building a human resource system that can manage and process massive data. The development of networking has made the amount of data grow exponentially, and the traditional processing methods have become inadequate. The industry has put forward the concept of big data and has studied related processing algorithms, etc., which has effectively improved data processing capabilities. The process of human resource management involves a large number of application requirements for processing massive data. Cloud computing platforms use their processing capabilities for big data to provide support for user applications. The value is simulated in Figure 5.

**Figure 6:**
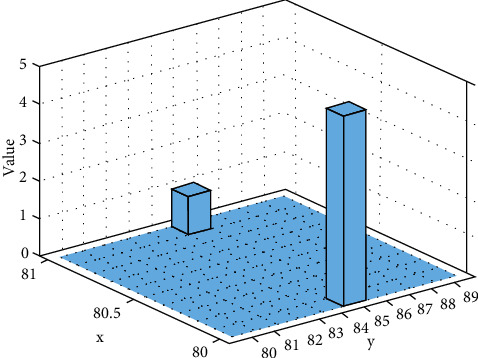
Value variation.

The system is divided into four functional modules, namely, tenant management, personnel information management module, attendance management module, and data analysis management module. The personnel data management module is mainly a massive data storage platform based on cloud computing, which completes the storage and rapid processing of all personnel data in the countryside. The main functions of this module include the function of customizing the personnel data template, which is used to determine the content of personnel data collection according to the actual needs of the countryside; the automatic audit function, which judges whether the personnel data is correct according to the logic rules; the self-service input function, which is used for personnel to pass the system customer self-service input of information and submission of information on the terminal; configuration of resource sharing functions to set the accessible scope of personnel information, etc. The data analysis module mainly uses the massive data parallel processing technology provided by the cloud computing platform to realize in-depth mining and analysis of human resource data. The main functions of this module include data query function, which is used to quickly complete the query of massive data according to user settings; summary statistics function, which is used to summarize the data according to certain requirements and display it to the user by visual methods such as charts, report management function, and export data processing results as tables. Tenant management is mainly used to manage system tenants, which can increase or decrease system tenants. In addition, this module can also be used to manage the basic information of each tenant and manage the rights management of each tenant to each module. The value variation is shown in Figure 6.

Different from the traditional dedicated system, the human resource management system based on the cloud computing platform provides related services for multiple enterprises through the SaaS model. SaaS can be divided into four levels of maturity models. The first level is customized development. The cloud computing service provider develops a completely independent system for each tenant and is responsible for system upgrade and optimization. The cost is huge; the second level is configurable type, the service provider provides a set of the same system code for each tenant and provides differentiated services through configuration according to the needs of different tenants, and the maintenance cost is high; the third level is high-performance multitenant type, the service provider provides the same set of system instances for different tenants and provides the data isolation function between different tenants; the fourth level is the scalable multitenant type; this architecture is to solve the service performance problem when a large number of tenants participate. With the load balancing function, multiple system use cases are built, loads are reasonably distributed, and services are provided for users. According to the research objectives of this paper, it is decided to choose the SaaS architecture of the third-level maturity model.

**Figure 7:**
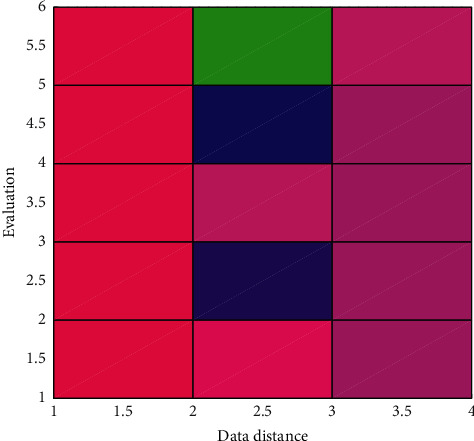
The evaluation.

The human resource management system based on the cloud computing platform can be logically divided into four layers, namely, the SaaS application presentation layer, the SaaS application logic layer, the application support platform layer, and the virtual infrastructure layer. Among them, the application presentation layer provides an operation interface for tenants, through which users complete functions such as user data entry, attendance and salary rule upload, and data processing result acquisition. This layer is also responsible for user single sign-on processing and forwarding of application requests. The evaluation is shown in Figure 7.

The application business logic layer is mainly to deploy specific business components. The business components adopt a modular development method. According to the identification carried by the tenant when applying, business processing is completed according to the configuration rules. This system includes personnel data entry, attendance information, automatic salary calculation, data statistical processing, and other business components. The components in this layer are in the form of services, which are called the upper-layer components. At the same time, in order to meet the tenant's personalized system, this layer also provides tenant configuration services.

**Figure 8:**
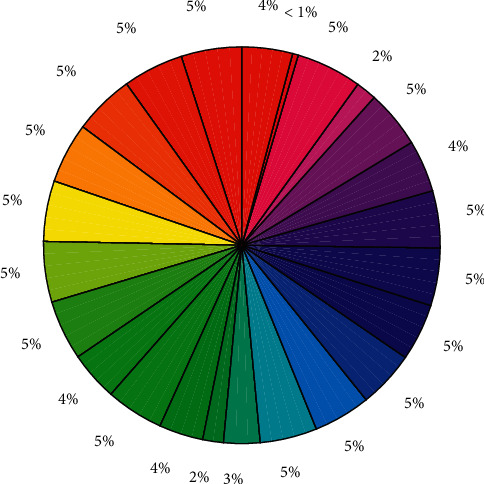
Percentage.

The application support layer mainly provides operation and construction support for the human resource system. Among them, operation support mainly provides information integration support, data optimization support, parallel computing framework support, etc. according to the needs of functional modules; construction environment support mainly provides development environment, storage model, computing model, etc. In the construction of this system, the components of the human resource data processing model and the framework of parallel computing belong to this support layer. The percentage is shown in Figure 8.

The virtual infrastructure layer mainly uses the virtualization technology in cloud computing to virtualize physical node resources located in different geographical locations and other computing resources into a dynamically adjustable resource platform to ensure that the functions of the system can be smoothly branched. In this system, this layer includes hardware facilities, network resources, database management systems, etc. of cloud computing service providers.

The cloud computing platform plays a fundamental role in the system cloud architecture design. The cloud computing platform has strong versatility for various processing methods of massive data. According to the purpose of data processing, the processing method is constructed and then combined with the specific application background; the data is deeply analyzed.

Thus, the system has the following function:

1. Analysis of the drawbacks of the traditional human resources system is mainly based on the design of a single rural entity, which cannot be satisfied with the management and analysis of the traditional human resources system, and the data cannot be shared, so that the rural areas cannot obtain beneficial results from the human resources data of various provinces and cities. Manage optimization programs. As the starting point, this paper analyzes the business needs of rural human resource personnel management personnel, attendance management personnel, and salary management personnel. Combined with performance indicators, the demand analysis of the rural human resource management system based on cloud computing is proposed.
2. According to the demand analysis, through the software-as-a-service model, the overall framework of the rural human resource management system based on cloud computing is designed and constructed, which mainly includes basic resource layer, data layer, software component layer, business logic layer, and service layer system structure, including personnel basic information management module, personnel attendance management module, personnel salary management module, and data statistics module. Through configurable data management technology, it can help rural areas to solve the problem of inconsistency in the basic information storage format of personnel in various towns and cities; through the configuration method of attendance rules, it can help rural areas to solve the problem of personalized attendance, set up salary and attendance management models, and realize the function of salary calculation and distribution; through the parallel computing method, the functions of big data aggregation, statistics, and analysis are realized.
3. According to the system design scheme, the GEA cloud computing platform is adopted to realize the main functions of the rural human resource management system based on cloud computing. The main technologies used are HBase, which is used to store basic information of rural personnel and improve the efficiency and speed of data operations; XML configuration file technology is used to realize the configurability of attendance rules; information integration technology is used to realize the automation of payroll processing automation; use Map/Reduce technology to realize big data processing.
4. By analyzing the actual application effect of the system, monitoring the system function indicators and system performance indicators, it is proved that the system functions and performance have achieved the expected goals.

## 5. Conclusion

The human resource information management system based on “cloud computing” is an important application and attempt of multidisciplinary technology in human resource management. The system runs stably, realizes extensive collaboration of data resources, and utilizes “cloud computing” resources. The integration advantages, collaborative management advantages, and distributed computing advantages realize the integrated processing of multisource data and meet the needs of different types of managers for human resource information management.

Continue to learn and apply cloud computing technology, improve the performance and service quality of cloud computing platform, and provide better services for more enterprises. Cloud computing platforms and big data analysis and processing technologies are constantly developing, and the application fields are also expanding. The improvement of existing methods to enable them to better handle massive data is the main direction of future development.

## Data Availability

The data used to support the findings of this study are available from the corresponding author upon request.
